# Role of the intestinal microbiome in colorectal cancer surgery outcomes

**DOI:** 10.1186/s12957-019-1754-x

**Published:** 2019-12-02

**Authors:** Lelde Lauka, Elisa Reitano, Maria Clotilde Carra, Federica Gaiani, Paschalis Gavriilidis, Francesco Brunetti, Gian Luigi de’Angelis, Iradj Sobhani, Nicola de’Angelis

**Affiliations:** 1 Department of Digestive and Hepatobiliary Surgery, Henri Mondor University Hospital, Avenue du Maréchal de Lattre de Tassigny, 94010 Créteil, France; 2 EA 7375-EC2M3, Université Paris Est – UPEC, 51, Avenue du Maréchal de Lattre de Tassigny, 94010 Créteil, France; 3 Department of Odontology, Rothschild University Hospital, 5 Rue Santerre, 75012 Paris, and University Paris Diderot, 75006 Paris, France; 4 Gastroenterology and Endoscopy Unit, University Hospital of Parma, University of Parma, Via Gramsci 14, 43126 Parma, Italy; 5 Department of Hepato-Pancreato-Biliary and Liver Transplant Surgery, Queen Elizabeth University Hospitals Birmingham NHS Foundation Trust, Birmingham, UK; 6 Department of Gastroenterology, Henri Mondor University Hospital, Avenue du Maréchal de Lattre de Tassigny, 94010 Créteil, France

**Keywords:** Intestinal microbiome, Colorectal cancer, Surgical outcomes, Oncological outcomes, Systematic review

## Abstract

**Objectives:**

Growing evidence supports the role of the intestinal microbiome in the carcinogenesis of colorectal cancers, but its impact on colorectal cancer surgery outcomes is not clearly defined. This systematic review aimed to analyze the association between intestinal microbiome composition and postoperative complication and survival following colorectal cancer surgery.

**Methods:**

A systematic review was conducted according to the 2009 PRISMA guidelines. Two independent reviewers searched the literature in a systematic manner through online databases, including Medline, Scopus, Embase, Cochrane Oral Health Group Specialized Register, ProQuest Dissertations and Theses Database, and Google Scholar. Human studies investigating the association between the intestinal microbiome and the short-term (anastomotic leakage, surgical site infection, postoperative ileus) and long-term outcomes (cancer-specific mortality, overall and disease-free survival) of colorectal cancer surgery were selected. Patients with any stage of colorectal cancer were included. The Newcastle-Ottawa scale for case-control and cohort studies was used for the quality assessment of the selected articles.

**Results:**

Overall, 8 studies (7 cohort studies and 1 case-control) published between 2014 and 2018 were included. Only one study focused on short-term surgical outcomes, showing that anastomotic leakage is associated with low microbial diversity and abundance of *Lachnospiraceae* and *Bacteroidaceae* families in the non-cancerous resection lines of the stapled anastomoses of colorectal cancer patients. The other 7 studies focused on long-term oncological outcomes, including survival and cancer recurrence. The majority of the studies (5/8) found that a higher level of *Fusobacterium nucleatum* adherent to the tumor tissue is associated with worse oncological outcomes, in particular, increased cancer-specific mortality, decreased median and overall survival, disease-free and cancer-specific survival rates. Also a high abundance of *Bacteroides fragilis* was found to be linked to worse outcomes, whereas the relative abundance of the *Prevotella*-co-abundance group (CAG), the *Bacteroides* CAG, and the pathogen CAG as well as *Faecalibacterium prausnitzii* appeared to be associated with better survival.

**Conclusions:**

Based on the limited available evidence, microbiome composition may be associated with colorectal cancer surgery outcomes. Further studies are needed to elucidate the role of the intestinal microbiome as a prognostic factor in colorectal cancer surgery and its possible clinical implications.

## Background

In recent years, several studies have shown the impact of the intestinal microbiome on host health and disease development. The human intestinal microbiome is a complex community of bacteria, archaea, viruses, and eukaryotes that is subject-specific and stable in healthy individuals [1]. Disturbances in the balance of the composition and function of the microbiome are associated with the onset of various pathologies, including obesity, Crohn’s disease, and gastrointestinal malignancies [2]. Indeed, a growing amount of evidence supports the role of the microbiome as a risk factor for the carcinogenesis of several malignancies, including colorectal cancers [3]. Conversely, the impact of the microbiome on the occurrence of postoperative complications and on the development of local recurrence after colorectal cancer surgery is not clearly defined.

Assessing whether the microbiome is a potential risk factor for postoperative complications of colorectal surgeries could lead to modifying perioperative care, as multiple perioperative interventions, such as mechanical bowel preparation (MBP) and antibacterial therapy (ABT), drastically influence microbiome composition, especially bacterial diversity [4, 5]. MBP, with or without ABT, is widely but empirically implemented before colorectal surgery to carry out a “clean” intervention that minimizes the risk of fecal contamination of the operative field, particularly during the anastomosis preparation. However, it has been shown that the loss of bacterial diversity could be a risk factor for postoperative complications, questioning the role of preoperative MBP and ABT [4].

Studies of animal models have shown that a eubiotic state is important for normal wound healing, including anastomosis repair after colorectal surgery [6]. Bacterial competition and cooperation can either promote or hamper wound healing during the inflammatory phase, influencing cellular activation and fibrosis in the wound repair process [5]. In the eubiotic state, bacteria remain harmless and do not cause infections, whereas following changes in the local environment and the induction of dysbiosis, by surgical injury for example, bacterial invasion and tissue inflammation take place [7].

Due to the major role of the microbiome in inflammation and wound healing [8], it is plausible that dysbiosis may be related to the development of colorectal surgery complications, such as anastomotic leakage (AL), surgical site infections (SSI), and prolonged postoperative ileus (PPI) [9, 10].

The composition of the intestinal microbiome may also impact the long-term outcomes of surgical treatments of colorectal cancer. Local recurrences of colorectal cancer are reported in 1–23% of cases after curative surgery [11]; this rate varies depending on tumor stage, localization, neoadjuvant and adjuvant therapies, and surgical technique [12]. Moreover, the occurrence of major postoperative complications has been related to worse oncological outcomes and overall and disease-free survival [13]. Of note, regarding the occurrence of postoperative complications, the administration of adjuvant chemotherapy may be delayed or contraindicated due to an insufficient patient performance status, which impacts prognosis [14].

In this context, it would be important to characterize the intestinal microbiome not only as a potential contributor to early postoperative complications (e.g., AL) but also as a potential marker of cellular and molecular mechanisms linked to local recurrence.

The present systematic review aims to analyze the available literature about the intestinal microbiome and its association with postoperative complications and long-term oncological outcomes after colorectal cancer surgery. To our knowledge, there is no existing review on the topic that has been performed with a systematic approach.

## Materials and methods

### Data sources and search strategy

A systematic review was performed following the Cochrane collaboration-specific protocol [15] and was reported according to the Preferred Reporting Items for Systematic Reviews and Meta-Analyses (PRISMA) statement.

Studies that investigated the association between the intestinal microbiome and surgical outcomes/postoperative complications and/or oncological outcomes/survival in colorectal cancer patients were searched in the following databases without date restrictions: Medline (through PubMed), Scopus, Embase, Cochrane Oral Health Group Specialized Register, ProQuest Dissertations and Theses Database, and Google Scholar. A specific research equation was used for each database, using the following keywords and MeSH terms: microbiome, microbiota, colorectal, surgery, surgical procedures, operative surgical procedures, general surgery, complications, anastomotic leak, surgical wound infection, surgical site infection(s), ileus, recurrence, mortality, survival, outcomes.

According to the PICOS schema, the following criteria were used for the literature search and selection:

- P, population: adult patients with colorectal cancer who underwent surgical resection. Any stage of colorectal cancer (according to the AJCC classification) was considered [16].
- I, intervention: analysis of the luminal or mucosa-associated microbiome from fecal samples or colorectal tissues. Both culture-dependent and genome sequencing methods were considered.
- C, comparisons: patients with and without postoperative complications and patients with different bacterial DNA loads in their microbiome samples.
- O, outcome(s): postoperative complications 90 days after surgery, including AL, SSI, and PI. Long-term outcomes comprised overall survival (OS), cancer-specific survival (CSS), disease-free survival (DFS), and recurrence-free survival (RFS).
- S, study design: randomized and non-randomized clinical trials, including cohort and case-control studies.

Studies that investigated colorectal diseases other than cancer, evaluating the impact of probiotic treatments, or focusing exclusively on the pathogenesis of colorectal cancer were not eligible for inclusion. The search was limited to human studies published in English. Then, the literature review was completed by using the “related articles” function in PubMed, to ensure an extensive approach. Moreover, the reference lists of the eligible records and pertinent review articles that were not included in this study were double-checked to identify potential additional articles for inclusion.

The literature search and selection were performed by two independent reviewers (LL and ER). Records were removed from the selection if both reviewers excluded the articles at the title/abstract screening levels. Disagreement was resolved via a discussion with a third reviewer (NdeA). Overall, the concordance rate between the two reviewers was 95%.

Details of the study protocol are registered in the International prospective register of systematic reviews, PROSPERO (ID number CRD42019117597).

### Data extraction

Both reviewers performed an independent full-text analysis and data extraction by filling in an electronic database. Extracted data included the first author’s name, year of publication, number of patients, type of microbiome, type of postoperative complications (AL, SSI, PPI), and oncological outcomes (recurrence, OS, DFS). Characterization of both the luminal microbiome (LM, fecal microbiome) and the mucosa-associated microbiome (MAM) was considered and described [17].

AL is defined as a defect in the bowel wall at the anastomotic site (including suture and staple lines of neorectal reservoirs) leading to communication between the intraluminal and extraluminal compartments [18].

SSI refers to an infection that develops 30 days after the surgery (or 1 year after the surgery if an implant has been placed) and is classified as superficial incisional SSI, deep incisional SSI, and organ/space SSI [19].

PPI is generally characterized by the presence of nausea and vomiting, inability to tolerate oral dietary intake, abdominal distension, and delayed passage of flatus and stool during the postoperative period [20].

### Study quality assessment and risk of bias

Two reviewers (LL and ER) carried out the study quality assessment and risk of bias evaluation of the selected articles. According to the study design, the New Castle–Ottawa scale (NOS) was used [21].

## Results

### Literature search and selection

**Fig. 1:**
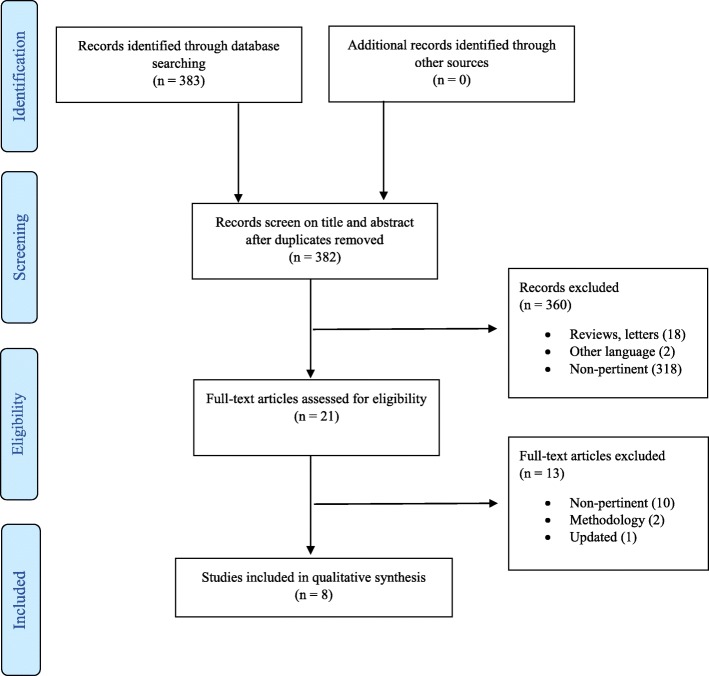
PRISMA flow diagram for study search, selection, inclusion, and exclusion. Example or search terms strategy: (("microbiota"[MeSH Terms] OR "microbiota"[All Fields] OR "microbiome"[All Fields]) AND colorectal[All Fields]) AND (("surgery"[Subheading] OR "surgery"[All Fields] OR "surgical procedures, operative"[MeSH Terms] OR ("surgical"[All Fields] AND "procedures"[All Fields] AND "operative"[All Fields]) OR "operative surgical procedures"[All Fields] OR "surgery"[All Fields] OR "general surgery"[MeSH Terms] OR ("general"[All Fields] AND "surgery"[All Fields]) OR "general surgery"[All Fields]) OR ("complications"[Subheading] OR "complications"[All Fields]) OR ("anastomotic leak"[MeSH Terms] OR ("anastomotic"[All Fields] AND "leak"[All Fields]) OR "anastomotic leak"[All Fields]) OR ("surgical wound infection"[MeSH Terms] OR ("surgical"[All Fields] AND "wound"[All Fields] AND "infection"[All Fields]) OR "surgical wound infection"[All Fields]) OR ("ileus"[MeSH Terms] OR "ileus"[All Fields]) OR ("recurrence"[MeSH Terms] OR "recurrence"[All Fields]) OR ("mortality"[Subheading] OR "mortality"[All Fields] OR "mortality"[MeSH Terms]) OR ("mortality"[Subheading] OR "mortality"[All Fields] OR "survival"[All Fields] OR "survival"[MeSH Terms]) OR outcomes[All Fields])

The initial search yielded 383 results; after removing duplicates, 382 articles were screened for eligibility based on title and abstract, and 21 articles were retrieved for a full-text evaluation. A total of 8 studies fulfilled the inclusion criteria and were finally included in the review [22–29] (Fig. 1). The excluded articles and detailed reasons for exclusion are reported in Additional file 1: Table S1.

### Study characteristics

The selected studies were published between 2014 and 2018, and there were 7 cohort studies [22–28] and 1 case-control [29]. The studies were carried out in Europe (*n* = 3), North America (*n* = 2), and Asia Pacific (*n* = 3). The general characteristics of the studies examined are summarized in Table 1. The overall total number of patients considered is 3263.

**Table 1:** Characteristics of the included studies

Authors, year	Study design	No. of patients^1^	Tumor stage	Microbiome type, tissue sample	Detection method	Bacteria^2^	Short-term surgical outcomes	Long-term oncological outcomes	Mean (SD) or median (range) follow-up	Adjustments on covariates
Flanagan et al., 2014	Cohort	32	Dukes staging A-D	MAM, tumor	Bacterial DNA, qPCR	*Fusobacterium nucleatum*	Not reported	• Overall survival	5 years	Not specified
Flemer et al., 2018	Cohort	47	AJCC I-IV	MAM, tumor	Bacterial DNA, V3-V4 16S rRNA	CAG	Not reported	• Overall survival	1371 days (67–1792 days)	Tumor stage, age, gender, treatment with chemotherapy and/or radiotherapy and cancer site
Kosumi et al., 2018	Cohort	1313	AJCC I-IV	MAM, tumor	Bacterial DNA, qPCR	*Bifidobacterium*	Not reported	• Cancer-specific mortality • Overall mortality	14.3 years (10–18.3 years)	Microsatellite instability status, CpG island methylator phenotype, long-interspersed nucleotide element-1 methylation, KRAS, BRAF, and PIK3CA mutations.
Mima et al., 2016	Cohort	1069	AJCC I-IV	MAM, tumor	Bacterial DNA, qPCR	*Fusobacterium nucleatum*	Not reported	• Cancer-specific mortality • Overall mortality	10.7 years (7–15.8 years)	Age, sex, year of diagnosis, family history of colorectal carcinoma in a first-degree relative, tumor location, microsatellite instability (MSI, mismatch repair deficiency), CpG island methylator phenotype (CIMP), KRAS, BRAF, and PIK3CA mutations, and LINE-1 hypomethylation (low-level methylation).
Van Praagh et al., 2017	Case-control	118	Not specified^3^	MAM, anastomosis	Bacterial DNA, V3-V4 16S rRNA	All	Anastomotic leakage	Not reported	Not reported	Not specified
Wei et al., 2016	Cohort	180	AJCC I-IV	MAM, tumor	Bacterial DNA, V4 16S rRNA	All	Not reported	• Overall survival • Disease-free survival	47 months (36–59 months)	Not specified
Yan et al., 2017	Cohort	208	AJCC III-IV	MAM, tumor	Bacterial DNA, qPCR	*Fusobacterium nucleatum*	Not reported	• Cancer-specific survival • Disease-free survival	Not reported	Not specified
Yu et al., 2017	Cohort	296	AJCC II-III	MAM, tumor	Bacterial DNA, qPCR	*Fusobacterium nucleatum*	Not reported	• Recurrence-free survival	Not reported	Not specified

*AJCC*, American Joint Committee on Cancer; *MAM*, mucosa-associated microbiome; *CAG*, tissue-associated microbial co-abundance groups; *qPCR*, quantitative polymerase chain reaction

^1^Patients included in the analysis of the microbiome and its association with short- or long-term outcomes

^2^Bacteria analyzed for an association with short- or long-term outcomes

^3^The study also included 6 non-oncological patients

Of the 8 selected articles, only one analyzed short-term outcomes, more specifically, the rate of AL [29]. No data were reported concerning SSI or PI. The other 7 articles focused on oncological long-term outcomes, that is, overall survival, mortality, and recurrence.

All of the included studies used microbial DNA analysis as a method for microbiome detection and characterization. Three articles studied the general composition of the microbiome in the sample, while 5 others looked for a particular bacterium, as shown in Table 1. In the first three studies [23, 26, 29], culture-independent profiling of bacterial communities in tissue samples was performed using amplification of bacterial 16S ribosomal RNS gene (16S rRNS). While Flemer et al. [23] and Praagh et al. [29] targeted variable regions V3 and V4 of the gene, Wei et al. [26] sequenced only V4 region. In the studies of Wei et al. and Praagh et al., statistical analysis was performed on the family, genus, and species level, whereas Flemer et al. did genus-based analysis. Rest of the studies [22, 24, 25, 27, 28] focused on detection of specific bacterial genus—*Bifidobacterium* [24]—or species—*Fusobacterium nucleatum* (*Fn*) [22, 25, 27, 28]. DNA detection and bacteria quantification were performed by real-time quantitative PCR (qPCR) method targeting 16S rRNA gene DNA sequence of *Bifidobacterium* and different *Fn* genes (16 s rRNA gene, nusG gene). In all studies but one [29], differences in microbiome composition were analyzed according to tumor stage. The main results of the selected studies are summarized in Table 2.

**Table 2:** Summary of the main results of the included studies

Authors, year	Bacteria^1^	Bacterial characteristics in tissues	Association between microbiota composition and tumor stage	Results
Short-term outcomes				
Van Praagh et al., 2017	*Lachnospiraceae* *Bacteroidaceae*	High abundance + Low microbial diversity in patients with or without C-seal	Not reported	- AL patients without a C-seal showed a significant lower microbial diversity, more *Bacteroides*, more *Lachnospiraceae*, and less *Prevotella* and *Streptococci* than C-seal patients who developed AL. - AL cases of non-C-seal patients seem to be almost without exception dominated by *Lachnospiraceae* and *Bacteroidaceae* with correspondingly low microbial diversity scores - Relation between the composition of the intestinal microbiota and the subsequent development of AL after stapled colorectal anastomoses, but only in patients who underwent surgery without the additional C-seal that covered the anastomoses
Long-term outcomes				
Flanagan et al., 2014	*F. nucleatum*	High vs. low or fold increase from normal	No significant differences between patients with no/low or high *F. nucleatum*, in TNM/Dukes staging.	- A significant difference in survival between patients without detected *F. nucleatum* in tumor tissue or low fold increase vs. those with high fold increase. - Median survival of subjects with high *F. nucleatum* fold increase is 2 years, whereas all subjects with low tumor to normal ratio survive more than 3 years (HR = 19.96, 95% CI = 1.42–281.42, *p* = 0.0266).
Flemer et al., 2018	Pathogen *CAG* *Prevotella CAG* *Bacteroides CAG* *Firmicutes CAG*	Relative abundance	Not specified	- Pathogen CAG-type microbiota was associated with longer survival (HR = 0.8, CI = 0.6–1.06; *p* = 0.12) - Prevotella CAG-type microbiota was associated with longer survival (HR = 0.36, CI = 0.12–1.1; *p* = 0.075). - Bacteroidetes CAG was associated with longer survival (HR = 0.75, CI = 0.58–1.03; *p* = 0.078). - Firmicutes CAG 2 was associated with shorter survival (HR = 1.52, CI = 0.84–2.75; *p* = 0.17)
Kosumi et al., 2018	*Bifidobacterium*	Negative vs. low vs. high DNA weight	Difference in Bifidobacteria was not associated with disease stage	No significant associations of the amount of Bifidobacteria with colorectal cancer-specific mortality or overall mortality
Mima et al., 2016	*F. nucleatum*	High vs. low vs. negative DNA load	The amount of tissue *F. NUCLEATUM* DNA was associated with higher pT stage (*p* = 0.0007). The association was not statistically significant with pN or M stage.	- Compared with *F. nucleatum*-negative cases, *F. nucleatum*-high cases had an HR = 1.58 (95% CI = 1.04–2.39) for cancer-specific mortality - A higher amount of tissue *F. nucleatum* DNA was associated with shorter colorectal cancer-specific survival (*p* = 0.023) but no difference in overall mortality rate
Wei et al., 2016	*B. fragilis* *F. nucleatum* *F. prausnitzii*	High vs. low abundance	- High abundance of *F. nucleatum* was significantly correlated with positive lymph node metastasis - High abundance of *F. prausnitzii* and *F. nucleatum* was significantly correlated with worse depth of invasion	- Higher level of *B. fragilis* (9.75% vs. 2.62%, FDR = 0.017) in non-survival group than in survival group, - *F. prausnitzii* (2.96% vs. 0.92%, FDR = 0.028) and *Methylobacterium suomiense* (1.91% vs. 0.78%, FDR = 0.098) were more abundant in the survival group. - *F. nucleatum* was higher in non-survival group than survival group (5.66% vs. 1.08%, FDR = 0.076) and it exhibited a greater abundance in the recurrence group than in survival group (5.10% vs. 1.08%, FDR = 0.08) - *B. fragilis* and *F. prausnitzii* might be correlated with patient’s survival in CRC - 3-year OS was significantly lower in patients with high *B. fragilis* and *F. nucleatum* than in those with low abundance of these two microbiota (*p* = 0.001, *p* = 0.003). - Low abundance of *F. prausnitzii* showed worse 3-year OS, (*p* = 0.06). - *B. fragilis* (HR = 2.01; 95% CI = 1.02–3.96; *p* = 0.044) and *F. nucleatum* (HR = 1.99; 95% CI = 1.02–3.87; *p* = 0.042) were independent predictor of the 3-year OS - *B. fragilis* (HR = 2.04; 95% CI = 1.11–3.73; *p* = 0.021) and *F. nucleatum* (HR = 1.82; 95% CI = 1–3.34; *p* = 0.05) were associated with poor 3-year DFS both
Yan et al., 2017	*F. nucleatum*	High vs. low level	- In both stage III and IV tumor, *F. nucleatum* level was significantly higher in CRC tissues than in adjacent normal tissues - *F. nucleatum* was found to significantly associated with tumor invasion (*p* = 0.015), LNM status (*p* = 0.008), and distant metastasis (*p* = 0.020). - Stage IIIA patients with low *F. nucleatum* level had no better CSS and DFS than those with high *F. nucleatum* level - High *F. nucleatum* level was significantly associated with worse CSS and DFS in stage IIIB and IV patients	- Patients with high *F. nucleatum* level had a significantly worse CSS and DFS than those with low *F. nucleatum* level For CSS: HR = 2.22; 95% CI = 1.48–3.32; *p* < 0.001 For DFS: HR = 2.0; 95% CI = 1.39–2.86; *p* < 0.001
Yu et al., 2017	*F. nucleatum*	High vs. low amount	The amount of *F. nucleatum* was positively associated with the AJCC stage and tumor size	- The 5-year RFS was substantially shorter in the *F. nucleatum*-high group than the *F. nucleatum*-low group. - *F. nucleatum* was an independent predictor of CRC aggressiveness with significant HR for predicting clinical outcome. Its predictive value was comparable with that of the AJCC stage

*AL*, anastomotic leakage; *CAG*, tissue-associated microbial co-abundance groups; *CRC*, colorectal cancer; *HR*, hazard ratios; *OS*, overall survival; *CSS*, cancer-specific survival; *DFS*, disease-free survival; *RFS*, recurrence-free survival; *NS*, not stated in the manuscript; *LNM*, lymph node metastasis

^1^Bacteria specifically associated with analyzed outcome

### Microbiome and short-term outcomes

Only one study published by van Praagh et al. investigated the relationship between a short-term surgical outcome, namely AL, and the composition of the intestinal microbiome [29]. The article reports a secondary analysis of data obtained from a previously published study [30] and investigates the association of intestinal microbiome with the development of colorectal AL depending on treatment method. The authors analyzed AL that required reintervention in patients who underwent colorectal surgery with or without the placement of a C-seal, a bioresorbable sheath stapled to the anastomosis. The mucosa-associated microbiome was analyzed from the stapled colorectal “donut.” The results of this study showed that in the patient group without the C-seal, AL was significantly associated with a lower biodiversity of the microbiome and a higher abundance of the mucin-degrading microbiome families *Lachnospiraceae* and *Bacteroidaceae* compared with matched patients who did not develop AL. Conversely, in the patient group receiving the C-seal, who showed a slightly higher rate of AL than the non-C-seal group, no association between AL and microbiome composition was detected. Only a few opportunistic pathogenic groups with a low abundance were associated with AL, particularly *Prevotella oralis.* The authors concluded that in patients who underwent colorectal surgery without C-seal placement, a bacterial composition of the intestinal microbiome consisting of 60% or more *Lachnospiraceae* and *Bacteroidaceae* is a predictive factor for AL. The lack of this association in C-seal patients may be explained by the effect of C-seal placement, which creates a new ecosystem that consequently influences and alters the composition of the resident microbiome [29]. Although the largest proportion of patients had colorectal cancer, it is worth noting that 3 patients in each group underwent surgery for non-oncological colorectal diseases.

### Microbiome and long-term outcomes

High heterogeneity was found in terms of considered outcomes and detected bacteria in studies evaluating long-term outcomes of colorectal cancer surgery in relation to intestinal microbiome.

The bacterium most frequently studied in cancer samples is *Fusobacterium nucleatum* [22, 25–28] (*F. nucleatum*). Studies have shown that *F. nucleatum* abundance increases as the disease progresses from an adenoma to cancer [22]. Levels of *F. nucleatum* are found to be higher in higher pT stages of cancer [25]. However, a study by Flanagan et al. found discordant results showing no differences between *F. nucleatum* levels and tumor stage or mutation status of KRAS and BRAF [22]. All studies investigating the role of *F. nucleatum* confirmed that higher levels of *F. nucleatum* in tumor samples correlated with worse outcomes in terms of OS, DFS, or cancer-specific survival [22, 25–28], with hazard ratios ranging from 1.58 to 19.96 (Table 2). An unfavorable prognosis could also be linked to the fact that *F. nucleatum* helps to activate autophagy-related pathways in colorectal cancer patients, promoting chemoresistance to oxaliplatin and 5-FU [28].

One study investigated the concentrations of *Bifidobacterium*, whose presence in tumor samples has been linked to the presence of signet ring cells [24]. In this study, Kosumi et al. reported that *Bifidoacterium* was found in 30% of the tumor samples, but no statistically significant correlation was noted in terms of the immune response or in the survival analysis (considering both cancer-specific survival and overall mortality).

Another study investigated the co-abundance groups (CAGs) of different bacteria [23]. The *Prevotella* CAG, pathogen CAG, and *Bacteroidetes* CAG were associated with improved survival. Although the presence of these specific CAGs has been linked to inflammatory response activation and high levels of *Prevotella*, pathogen CAGs were shown to positively influence cancer-related survival. Other bacteria, such as *Faecalibacterium prausnitzii*, were correlated with a better survival outcome [26].

### Study quality assessment

Based on the Newcastle-Ottawa Quality assessment scale (NOS), one study received 8/9 stars, three studies received 6/9, three studies received 5/9, and one study received 4/9. The details are displayed in Additional file 1 Table S2. It must be stressed that a high heterogeneity in the study designs, study populations, analyzed microbiomes, and outcomes was observed. All included studies were retrospective cohort or case-control studies.

## Discussion

The present systematic review is the first, to our knowledge, to analyze the existing literature about the intestinal microbiome and its association with short- and long-term outcomes after colorectal cancer surgery in a systematic manner.

Literature about the impact of microbiome diversity on the occurrence of postoperative complications after colorectal cancer surgery is very limited. Only one study focused on the relationship between the composition of the microbiome and AL [29] and showed that a low microbial diversity and high abundances of *Lachnospiraceae* and *Bacteroidaceae* were significantly related to AL. These findings were used for a predictive analysis, which revealed that samples with a total sum of *Lachnospiraceae* and *Bacteroidaceae* higher than 60% and a Simpson diversity score on the family level < 0.75 are prone to developing AL, with an odds ratio of 28 (95% confidence interval not reported). Interestingly, both bacterial families are normal habitants of the intestinal microbiome and are important for the healthy functioning of the colonic epithelium, with *Faecalibacterium prausnitzii* and *Eubacterium rectale* (*Lachnospiraceae* family) being the two most common species in the human gut [31]. The *Lachnospiraceae* family is rich in butyrate-producing bacteria that provide a crucial energy source for colon epithelial cells that is essential for the maintenance of gut barrier functions and immunomodulatory and anti-inflammatory properties. To explain the findings with possible pathophysiological effects, it would be crucial to compare the composition of the microbiome before and after surgery.

Half of the included studies focused on *F. nucleatum* and its association with the long-term oncological outcomes of colorectal cancer patients. All studies showed that high concentrations of *F. nucleatum* had a negative impact on survival outcomes and were associated with decreased OS [26], decreased median survival [22], decreased CSS [27], decreased DFS [26, 27], decreased RFS [28], and increased cancer-specific mortality [25]. However, we must be careful in interpreting these results because the available data rarely or only partially considered the many potential confounding factors that can influence survival outcomes.

In recent years, multiple reviews have analyzed the relationship between *Fusobacteria* and colorectal cancer. Several studies that detected a higher abundance of *F. nucleatum* in colorectal adenomas compared with healthy colorectal tissues [32] suggested the possible involvement of this bacterium in the pathogenesis of colorectal cancer. A study by Flanagan et al. [22] supports this hypothesis, indicating that not only there are higher *F. nucleatum* levels in individuals with adenomas than in controls but also that *F. nucleatum* levels increase through adenomatous stage progression from a tubular adenoma to a high-grade dysplasia and colorectal cancer. Indeed, *F. nucleatum* is able to invade human epithelial cells, activate the β-catenin pathway, and trigger oncogenic gene expression using the FadA adhesion virulence factor; consequently, it can stimulate colorectal cancer cell growth [33]. Moreover, a high abundance of *F. nucleatum* is associated with cancer progression and lymph node metastases, as shown in the study by Li et al. [34]. The majority of the included studies [25–28] also found a significant association of *F. nucleatum* with one or more unfavorable cancer characteristics—advanced stage, tumor invasion, lymph node metastasis, and tumor size. Further important evidence was obtained from the study by Yu et al. [28], which highlighted the negative impact of *F. nucleatum* on oncological outcomes. This study compared microbiome composition between recurrent and non-recurrent colorectal cancers and found that *F. nucleatum* is the most enriched bacterium in recurrent cases. Moreover, the study suggests that *F. nucleatum* may promote resistance to oxaliplatin and 5-FU chemotherapy regimens via the colorectal cancer cell autophagy pathway, therefore leading to decreased recurrence-free survival.

Another bacterium that was found to be associated with unfavorable oncological outcomes was *B. fragilis*, as shown in the study by Wei et al. [26]. Like *F. nucleatum*, *B. fragilis* was also associated with decreased 3-year OS and DFS. However, unlike *F. nucleatum*, which is an indigenous species in the oral cavity and is associated with only pathological changes in the colon and rectum, *B. fragilis* is present in the intestinal microbiome of all healthy individuals. In particular, enterotoxigenic *B. fragilis* (ETBF) has been linked to the development of colorectal cancer. Purcell et al. [35] showed an increased abundance of ETBF in early stage carcinogenic lesions—low-grade dysplasia and tubular adenomas. Viljoen et al. [36] described significantly higher ETBF and *F. nucleatum* levels in individuals with advanced stage III and IV colorectal cancer. Therefore, it remains to be determined whether the worse oncological outcomes described by Wei et al. [26] are due to specific pathways in carcinogenesis or the *B. fragilis* association with advanced cancer stages.

Kosumi et al. [24] analyzed the relationship between the *Bifidobacterium* genus and colorectal cancer characteristics and outcomes. *Bifidobacterium* has been of interest in many studies, mainly concerning probiotic administration and their effect on gastrointestinal and extra-digestive pathologies. *Bifidobacteria* are normal habitants of the intestinal tract and are present from an early age. The imbalance of *Bifidobacteria* has been associated with several non-oncological diseases [37], but few studies have suggested its association with colorectal cancer. Indeed, it has been shown that there are decreased levels of *Bifidobacteria* in patients with colorectal cancer compared with the levels in controls or patients with diverticular disease [38]. Conversely, Kosumi et al. [24] did not find any significant association between the amount of *Bifidobacteria* and cancer-specific and overall mortality or any relationship with the clinical, pathological, or molecular characteristics of colorectal cancer. Despite the paucity of literature, it is likely that *Bifidobacteria* exert a beneficial effect during the early stages of carcinogenesis, acting as a prebiotic agent in improving epithelial defenses against infections [39]. The study by Flemer et al. [23] is the only one that focused on the analysis and comparison of tissue-associated groups of various bacteria rather than individual bacteria. In their previous work [40], the authors defined 6 mucosa-associated bacterial co-abundance groups (CAGs) and showed an increased abundance of the *Firmicutes* CAG 2, the *Prevotella* CAG, the pathogen CAG, and the *Bacteroidetes* CAG 2 in tissues of colorectal cancer compared with their levels in tissues of control subjects. Moreover, they showed that the pathogen CAG and the *Prevotella* CAG were correlated and that their gene expression levels were previously associated with decreased survival in patients with colorectal cancer. In the most recent study, the results support the idea that the two latter CAGs are in fact associated with longer survival [23]. The authors stressed the importance of further studies to explain this outcome.

The present systematic review is based on 8 original studies with variable quality and risk of bias characterized by a high degree of heterogeneity in terms of study design, sample size, patient population, clinical characteristics, bacterial sampling and detection methods, target microbiome, and target outcome. Although all studies that focused on oncological outcomes detected MAM from tumor samples and in 5 of these studies [22, 25–28], they analyzed a DNA load of *F. nucleatum*, it must be stressed that each study used different types of samples. Only Flanagan et al. and Wei et al. used fresh frozen tissue samples as opposed to formalin-fixed paraffin-embedded (FFPE) samples used in the rest of the studies. It has been shown previously that FFPE specimens have lower expression of the analyzed targets than fresh frozen samples, which is of great importance in the quantitative analysis of bacteria [41]. Three studies [22, 23, 27] analyzed the microbiome in healthy tissues from the same patient as a reference. Although this approach is a common practice in studies focusing on microbiome detection in colorectal cancer and is used to exclude interindividual microbiome differences, there is contradictory evidence whether the healthy and cancerous tissues from the same subject are representative of healthy and cancer-related microbiome composition. Finally, it must be highlighted that the included studies were carried out in different geographical areas (e.g., Europe, Asia, the USA), a feature that may influence the diversity of the intestinal microbiome [42]. The mode of subsistence and diet seem to be the most important natural factors impacting the intestinal microbiome, and diet varies between ethnic groups, nationalities, and people who live in rural or urban areas, with the latter being especially enriched in Bacteroides, *Bifidobacterium*, and *Firmicutes* [42].

The limited number of studies and the methodological heterogeneity hampered any meta-analytic approach of the present data and it should be carefully considered when interpreting the available body of evidence.

### Clinical impact and future perspectives

Although the evidence remains limited, the potential relationship between intestinal microbiome composition and outcomes of colorectal cancer surgery is a promising field of research that may have compelling clinical relevance. Indeed, despite the significant advances in surgical techniques and devices, colorectal cancer surgery is still associated with a non-negligible morbidity and mortality risk. Concerning AL in particular, the role of microbiome composition may represent one missing piece to explain the susceptibility of certain patients. Indeed, detecting specific bacteria with a negative impact on anastomotic healing could help the surgeon adapting the surgical strategy (e.g., stoma vs. primary anastomosis) to the individual patient’s risk level, also taking into account the presence of an “unfavorable” microbiome. An experimental study of animal models has already shown successful attempts to prevent AL by introducing oral phosphate and phosphate carrier compounds after low colorectal anastomosis in the presence of pathogens expressing collagenase (*Pseudomonas aeruginosa*, *Serratia marcescens*, and *Enterococcus faecalis*) [43].

Another appealing concept regarding the intestinal microbiome is its possible use as a biomarker for the diagnosis of colorectal cancer, as well as its predictor of cancer aggressiveness, treatment outcomes, and even response to therapy. All included studies focusing on oncological outcomes suggested that specific bacteria such as *F. nucleatum* or CAGs could be used as independent predictors of survival, recurrence, and mortality in colorectal cancer patients. As a high abundance of *F. nucleatum* is associated with worse oncological outcomes of surgery, its detection in tumoral tissues before surgery could help to predict a more aggressive course of the disease, therefore leading to possible strategic changes in cancer treatment, such as more radical surgery with lymph node debulking, closer surveillance intervals for early recurrence detection, and even adjusted chemotherapy regimens.

In light of these clinical perspectives, we performed a quick search on *ClinicalTrial.gov* to identify pertinent ongoing clinical trials. We can report of two studies assessing the relationship between microbiome and postoperative complications (NCT04005118) or anastomotic leakage (NCT03496441) in patients undergoing colorectal surgery. Both studies are observation cohort studies; the first study is ongoing whereas the second one appears to have completed the recruitment phase. Their results will substantially contribute to elucidate the characteristics of the intestinal microbiome in patients developing or not postoperative complication after colorectal surgery and will support further clinical and animal research in the field. Indeed, it remains unclear whether microbiome composition is a cause or the result of clinical outcomes in colorectal cancer patients and its characterization is so complex and influenced by so many factors that will definitely require multiple investigations [44–46].

Moreover, researchers should continue to study the impact of preoperative ATB and MBP son intestinal microbiome and subsequently on the short- and long-term postoperative outcomes. Current evidence is still inconclusive about the efficacy of prophylactic techniques or preparations to avoid complications such as AL [5]. Thus, there is an urgent need for research studies to analyze the microbiome composition in deep, to elucidate the effects (potentially beneficial or harmful) of different microbes on the processes of wound healing and development of early postoperative complications [5, 47]. Finally, research may advance looking for treatments that may influence microbiome composition to favor symbiotic and beneficial microbes that can contribute to improve colorectal surgery outcomes.

## Conclusions

The present systematic review demonstrates that the current literature about the role of intestinal microbiome on colorectal cancer surgery outcomes is limited. However, there is consistency in the available data supporting a plausible linked between the intestinal microbiome composition and the occurrence of postoperative complications in colorectal cancer surgery patients. Further investigations are awaited to assess this association and elucidate whether intestinal microbiome characterization could be used as a prognostic marker in colorectal cancer patients.

## Supplementary information

**Additional file 1: Table S1.** Excluded articles and reasons for exclusion after full-text evaluation. **Table S2.** Risk of bias evaluation for cohort and case-control studies based on the Newcastle-Ottawa Scale. Maximum score for each item: Selection – 4, Comparability – 2, Outcome/Exposure – 3 stars

## Data Availability

All data and materials are available within the manuscript.
