# Supplementary material for: Role of the intestinal microbiome in colorectal cancer surgery outcomes
Source: World J Surg Oncol. 2019 Dec 2;17:204. doi: 10.1186/s12957-019-1754-x (PMC6889350; doi:10.1186/s12957-019-1754-x)
Supplement: Supplementary file 1 — Additional file 1: Table S1. Excluded articles and reasons for exclusion after full-text evaluation. Table S2. Risk of bias evaluation for cohort and case-control studies based on the Newcastle-Ottawa Scale. Maximum score for each item: Selection – 4, Comparability – 2, Outcome/Exposure – 3 stars [file 12957_2019_1754_MOESM1_ESM.docx]

Additional file

**Table S1. Excluded articles and reasons for exclusion after full-text evaluation**

| **First author** | **Title** | **Journal** | **Year** | **Reasons for exclusion** |
| --- | --- | --- | --- | --- |
| Liu Z et al. | Randomized clinical trial : the effects of perioperative probiotic treatment on barrier function and postoperative infectious complications in colorectal cancer surgery – a double blind study | Aliment Pharmacol Ther | 2011 | Not pertinent to the review question |
| Jeppsson B et al. | Use of probiotics as prophylaxis for postoperative infections | Nutrients | 2011 | Review, not pertinent to the review question |
| Aisu N et al. | Impact for perioperative probiotic treatment for surgical site infections in patients with colorectal cancer | Experimental and Therapeutic Medicine, | 2015 | Not pertinent to the review question |
| Hyspler R et al. | Markers of perioperative bowel complications in colorectal surgery patients | Disease markers | 2015 | Non-pertinent methodology |
| Mizuta M et al. | Perioperative supplementation with Bifidobacteria improves postoperative nutritional recovery, inflammatory response, and fecal microbiota in patients undergoing colorectal surgery: a prospective, randomized clinical trial | Biosci Microbiota Food Health | 2016 | Not pertinent to the review question |
| Van Praagh BJ et al. | Intestinal microbiota and anastomotic leakage of stapled colorectal anastomoses: a pilot study | Surg Endosc | 2016 | Updated in 2017, included in the review |
| Hibberd AA et al. | Intestinal microbiota is altered in patients with colon cancer and modified by probiotic intervention | BMJ Open Gastro | 2017 | Not pertinent to the review question |
| Sze, AM et al. | Normalization of the microbiota in patients after treatment for colonic lesions | Microbiome | 2017 | Not pertinent to the review question |
| Mima et al., | The role of intestinal bacteria in the development and progression of gastrointestinal tract neoplasm | Surg Oncol | 2017 | Review, not pertinent to the review question |
| Guthrie et al., | Human microbiome signatures of differential colorectal cancer drug metabolism | NPJ Biofilms Microbiomes | 2017 | Not pertinent to the review question |
| Gao et al.. | Gut microbioma and colorectal cancer | Eur J Clin Microbiol Infect Dis | 2017 | Not pertinent to the review question |
| Deng X et al. | Comparison of microbiota in patients treated by surgery or chemotherapy by 16S rRNA sequencing reveals potential biomarkers for colorectal cancer therapy | Frontiers in Microbiology | 2018 | Not pertinent to the review question |
| Polakowski BC et al. | Impact of the preoperative use of synbiotics in colorectal cancer patients: A prospective, randomized, double-blind, placebo-controlled study. | Nutrition. | 2019 | Non-pertinent methodology |

**Table S2. Risk of bias evaluation for cohort and case-control studies based on the Newcastle-Ottawa Scale.**

Maximum score for each item: Selection – 4, Comparability – 2, Outcome/Exposure – 3 stars

| **Criteria**  **Study** | **Selection** | **Comparability** | **Outcome/**  **Exposure** | **Overall score** |
| --- | --- | --- | --- | --- |
| Flanagan et al., 2014 | *** | - | ** | **5** |
| Flemer et al., 2018 | ** | ** | ** | **6** |
| Kosumi et al., 2018 | ** | * | *** | **6** |
| Mima et al., 2016 | *** | ** | *** | **8** |
| Praagh et al., 2017 | ** | - | *** | **5** |
| Wei et al., 2016 | *** | * | ** | **6** |
| Yan et al., 2017 | *** | * | * | **5** |
| Yu et al., 2017 | *** | - | * | **4** |
